# Using targeted next-generation sequencing to characterize genetic differences associated with insecticide resistance in *Culex quinquefasciatus* populations from the southern U.S.

**DOI:** 10.1371/journal.pone.0218397

**Published:** 2019-07-03

**Authors:** Linda Kothera, John Phan, Enas Ghallab, Mark Delorey, Rebecca Clark, Harry M. Savage

**Affiliations:** 1 Division of Vector-Borne Diseases, Centers for Disease Control and Prevention, Fort Collins, Colorado, United States of America; 2 Office of Advanced Molecular Detection (Scientific Computing), Centers for Disease Control and Prevention, Atlanta, Georgia, United States of America; Chinese Academy of Agricultural Sciences, CHINA

## Abstract

Resistance to insecticides can hamper the control of mosquitoes such as *Culex quinquefasciatus*, known to vector arboviruses such as West Nile virus and others. The strong selective pressure exerted on a mosquito population by the use of insecticides can result in heritable genetic changes associated with resistance. We sought to characterize genetic differences between insecticide resistant and susceptible *Culex quinquefasciatus* mosquitoes using targeted DNA sequencing. To that end, we developed a panel of 122 genes known or hypothesized to be involved in insecticide resistance, and used an Ion Torrent PGM sequencer to sequence 125 unrelated individuals from seven populations in the southern U.S. whose resistance phenotypes to permethrin and malathion were known from previous CDC bottle bioassay testing. Data analysis consisted of discovering SNPs (Single Nucleotide Polymorphism) and genes with evidence of copy number variants (CNVs) statistically associated with resistance. Ten of the seventeen genes found to be present in higher copy numbers were experimentally validated with real-time PCR. Of those, six, including the gene with the knock-down resistance (*kdr*) mutation, showed evidence of a ≥ 1.5 fold increase compared to control DNA. The SNP analysis revealed 228 unique SNPs that had significant p-values for both a Fisher’s Exact Test and the Cochran-Armitage Test for Trend. We calculated the population frequency for each of the 64 nonsynonymous SNPs in this group. Several genes not previously well characterized represent potential candidates for diagnostic assays when further validation is conducted.

## Introduction

Mosquitoes in the *Culex pipien*s complex are responsible for transmitting arboviruses that cause West Nile virus disease and St. Louis encephalitis. In the United States, the complex is widespread and its members are commonly found in close proximity to humans. The taxa included in the *Culex pipiens* complex in the U.S. include *Cx*. *pipiens* L., *Cx*. *quinquefasciatus* Say, and an uncommon autogenous form of *Cx*. *pipiens* known as *Cx*. *pipiens* form molestus Forskål. An epidemiologically relevant distinction among taxa is that *Cx*. *pipiens* form pipiens enters a state of diapause during the winter months, while *Cx*. *quinquefasciatus* and *Cx*. *pipiens* form molestus cannot be induced to diapause, even when given appropriate light cues [1–3]. Hybridization among the taxa occurs due to an absence of reproductive barriers, and a wide hybrid zone between *Cx*. *pipiens* and *Cx*. *quinquefasciatus* exists across much of the middle latitudes of the U.S. [4–8]. Hybridization between form molestus and form pipiens is less common, due to the paucity of form molestus populations in the U.S. [8–10].

Due to its close association with human-occupied habitats and capacity to transmit pathogens, *Culex pipiens* complex mosquitoes in the U.S. are often targeted for vector control. Treatments to reduce the numbers of adults and larvae are routinely used as part of control efforts. Targeting the adult stage (adulticiding) is an important control technique when virus-positive mosquitoes are discovered during surveillance activities, or when cases of human disease are reported.

The repeated use of insecticides can lead to resistance, where previously used amounts of product and frequencies of application fail to sufficiently reduce the number of adults in a population. Insecticide resistance is of particular concern during disease outbreaks, because insecticide resistance can impede the ability to control vector mosquitoes. Indeed, the development of resistance is hypothesized to have played a role in the rise of mosquito-borne diseases outside of the U.S. over the last several decades [11–14].

Insecticide resistance is thought to arise by two main mechanisms: 1) mutations to mosquito genes targeted by insecticides and 2) by increases in products made by detoxification genes, also known as metabolic resistance. A major and widespread type of resistance caused by target site mutations is referred to as *knock-down resistance* (*kdr*). Knock-down resistance results in insensitivity to pyrethroid insecticides and has been the frequent subject of resistance studies in the *Culex pipiens* complex [15–18]. The specific genes involved in *kdr* code for the Voltage Gated Sodium Channel (VGSC) and examples are so numerous that insecticide resistance is sometimes quantified solely in terms of whether or not a *kdr* mutation is present [19].

The most common *kdr* mutation in the *Culex pipiens* complex is known as L1014F, which causes an amino acid change from lysine to phenylalanine [20]. The number 1014 refers to the position of the relevant codon in the species where the mutation was first described, the house fly *Musca domestica* L. [21]. In the published *Culex quinquefasciatus* genome, the mutation is a TTA–TTT change that occurs in Exon 6, codon 406, of the gene CPIJ007595 [22, 23]. Prior to the publication of the genome, the L1014F mutation was described as being located on Exon 20 of the entire VGSC [24]. Additional mutations in *Culex pipiens* complex sodium ion channel genes have subsequently been discovered, and in some cases been shown to have additive effects on resistance [25, 26].

While the L1014F *kdr* mutation appears to be widespread in vector mosquito populations in the U.S., there is some evidence that defining resistance solely by its presence paints an incomplete picture of resistance. For example, a study by Yang and Liu [18] found the presence of the L1014F *kdr* mutation was insufficient to account for variability in levels of resistance and suggested additional mechanisms were likely present. Saavedra-Rodriguez et al. [27] derived a similar conclusion using QTL (Quantitative Trait Locus) mapping in another vector mosquito species, *Aedes aegypti* L. Thus resistance is perhaps best viewed as a complex phenomenon, capable of differing on the population level, where multiple genetic changes can contribute to a resistance phenotype [28–30].

The other well-documented gene with target site mutations affecting resistance in *Culex* mosquitoes is an acetylcholinesterase gene, CPIJ006034 [31]. Abbreviated *ACE-1*, such mutations confer resistance to organophosphate insecticides. Two *ACE-1* mutations, known as G119S and F290V (numbering following that in *Torpedo californica* Ayers [32]), either alone or in tandem can decrease mosquito sensitivity to organophosphate insecticides [33–35]. Synergistic effects have been demonstrated between the G119S *ACE-1* mutation and other mutations [36, 37].

The other major resistance mechanism, metabolic resistance, acts by increasing the amount of gene product made by detoxification genes. Such an increase can occur in two ways. First, there can be copy number variation (CNV) where a gene exists in the genome in multiple copies, also referred to as gene duplication, or gene amplification. Alternatively, a gene can be upregulated in resistant individuals, resulting in more product compared to that gene in susceptible individuals.

Examples of metabolic resistance via CNV in *Culex pipiens* complex include two esterase genes, CPIJ013917 and CPIJ013918, which confer resistance to organophosphate insecticides [38, 39]. Distinct alleles have been identified by their mobility during starch-gel electrophoresis and in the U.S., several alleles have been described based on mobility characteristics [40, 41].

Other upregulated genes implicated in metabolic resistance include cytochrome P450s (including monooxygenases; [42–44]) and glutathione S-transferases [45]. While both gene families have been known to be involved in resistance for some time, recent next generation sequencing studies have taken advantage of the published *Cx*. *quinquefasciatus* genome [46] and have begun to elucidate the specific genes involved. Such studies have largely compared the transcriptomes of resistant and susceptible colony strains in order to determine which genes are differentially expressed (and thus contribute to metabolic resistance) between the two phenotypes [47–52]. Recent work on other vector mosquitoes [53, 54] has shown an interesting association between the presence of certain SNP mutations and metabolic resistance, but to date such associations have not been established for *Culex*.

A transcriptome study by Yan et al. [55] compared the number of detoxification genes in the *Culex quinquefasciatus* genome to the number in the *Aedes aegypti* and *Anopheles gambiae* Giles genomes. Their work indicated the *Culex* genome contained more detoxification genes than the other species. They termed the additional genes “expansion” genes and hypothesized such genes might be involved in insecticide resistance.

Genomic sequence data for *Cx*. *quinquefasciatus* currently exist in the form of 3,172 supercontigs, and most genes have not been mapped to chromosomes, although several studies have mapped small numbers of genes [56–59]. Thus, inferences about linkage disequilibrium can be difficult to make at this time. Nevertheless, a gene set exists (Cpipj2.4) that was updated in October 2017 [23].

Routine surveillance that quantifies levels of insecticide resistance is beneficial because it can inform decisions about vector control. The objective of this study was to use targeted DNA sequencing to look for genetic differences between phenotypically susceptible and resistant *Culex quinquefasciatus* mosquitoes. Our goal is to expand the number and kinds of diagnostic molecular assays employed to characterize resistance as part of routine and outbreak vector surveillance. The use of DNA-based markers allows testing on specimens at any life stage and could augment existing efforts to assess levels of resistance obtained with bottle bioassays. To that end, we built upon the results of previous studies to develop a DNA targeted sequencing panel of detoxification genes known or hypothesized to be involved in the development of resistance, with some emphasis on the “expansion” genes described above, and sequenced them in resistant and susceptible individuals. We sought to characterize two kinds of genetic changes associated with insecticide resistance: mutations (SNPs) and CNVs.

## Materials and methods

### Specimen collection and resistance testing

**Fig 1:**
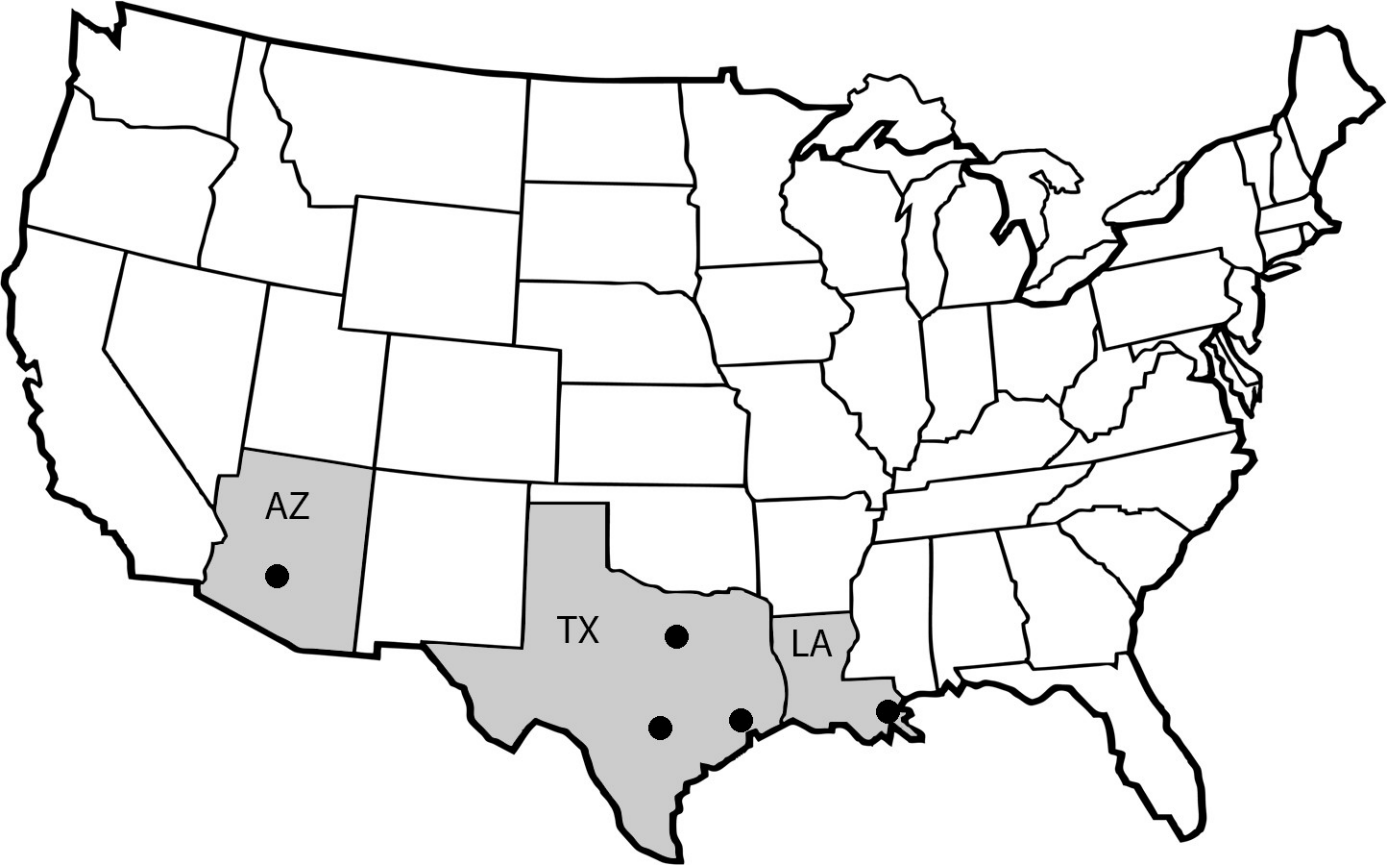
Map showing *Culex quinquefasciatus* egg raft collection sites in the southern U.S. See Table 1 for details. Reprinted under a CC BY license with permission from David S. Carlson, creator, original copyright 2018.

In the summers of 2014 and 2015, we worked with vector control districts to obtain specimens from eight *Cx*. *quinquefasciatus* populations in Arizona, Texas, and Louisiana (Fig 1, Table 1). Permission to access collection sites was granted previously to each control district in order to conduct routine mosquito surveillance activities. Several collection locations (HCT-HCU, NOT-NOU, TXT-TXU) were paired so that a “treated” site (one that was regularly treated with an adulticide) was located approximately 1–2 km from an “untreated” site (one not routinely treated with an adulticide). This was done to increase the chances that the specimens would share a genetic background and differences might be attributable to the application of insecticides, although specimens were grouped by phenotype for analyses. The remaining sites (AZU, WCU) were untreated with no corresponding treated site (Table 1). Approximately 40 egg rafts were collected from each population and the 20 largest families were ultimately used for insecticide resistance testing. Upon arrival to the CDC, each individually tubed egg raft representing one full sibling family was placed in a separate but identical plastic pan measuring 4.5 cm x 25 cm x 34 cm and containing approximately 500 ml of filtered water. Each pan received 50 ml of a 4 g/1 L liver powder (MP Biomedicals, Solon, OH) solution, and was covered with a sheet of acrylic so pans could be stacked. Pans were placed in incubators kept at 27.5° C and exposed to a 15:9 light-dark schedule. After three days, a small scoop (approximately 2g) of a mixture of finely ground fish food (Tetramin), liver powder and brain-heart infusion (Becton, Dixon and Co., Sparks, MD) was added to each pan. When approximately half of the specimens had pupated (usually after 4–5 days) one fourth instar larva was removed from each pan, examined with a dissecting scope and identified using a dichotomous key [60] to confirm that the raft progeny were *Culex quinquefasciatus*. If a different species was present, those specimens were frozen and discarded. Upon pupation, specimens were moved by family to individual cages measuring 20.3 cm on a side (Bioquip) and given 10% sugar water ad libitum. Insecticide resistance testing was performed on both male and female individuals aged 3–7 days.

**Table 1:** Collection information for *Culex quinquefasciatus* specimens used in this study.

Abbreviation	State	County or Parish	Treated or Untreated^a^	Latitude	Longitude	N^b^
AZU	Arizona	Maricopa	Untreated	33.32638	-111.93251	7
HCT	Texas	Harris	Treated	29.63225	-95.33457	16
HCU	Texas	Harris	Untreated	29.61398	-95.30950	14
NOT	Louisiana	New Orleans	Treated	30.03775	-89.91569	13
NOU	Louisiana	New Orleans	Untreated	29.96342	-90.06753	16
TXT	Texas	Dallas	Treated	32.84972	-96.79057	19
TXU	Texas	Dallas	Untreated	32.67312	-96.70780	19
WCU	Texas	Williamson	Untreated	30.63326	-97.67799	20

^a^Treated areas were defined as receiving regular adulticiding. Untreated areas were defined as those that had not received adulticiding treatments in recent memory.

^b^N = Number of families tested for resistance to malathion and permethrin insecticides. One individual per family was used for targeted DNA sequencing.

We tested each family for resistance to two insecticides, malathion and permethrin. The technical grade active ingredients were used (ChemService, West Chester, PA). The number of families tested per population ranged from 12–20 (N = 142). We followed the CDC bottle bioassay protocol [61] with slight modifications. Two bottles each of malathion and permethrin were prepared per family along with one control bottle that was treated with 1ml of acetone. Approximately 10–20 individual adult mosquitoes were aspirated into each bottle. Mosquitoes alive at the end of the test were considered highly resistant. Susceptible mosquitoes were defined as those that died at or before 30 minutes of exposure to permethrin or 45 minutes of exposure to malathion. When all individuals were dead, they were poured out of the bottles, placed with forceps into labeled tubes and stored at -20°C. If there were survivors at the end of the test (i.e. at 120 minutes) the dead individuals were carefully aspirated out of the bottles and placed into labeled tubes, then the survivors were immobilized with CO_2_ and treated as above. All specimens were subsequently stored at -80° C. Each family was then categorized as resistant (≤ 80% dead at the cut off time) or susceptible (more than 80% dead at the cut off time) to each insecticide. One specimen from each of 125 families tested for resistance was used for subsequent DNA sequencing and represented the phenotype for both insecticides. Not all families tested for resistance were subsequently sequenced, as there was a small number of families where the bottle bioassay results were different between the two bottles for an insecticide. S1 Table lists the resistance phenotypes for each bottle bioassay-tested family used in this study.

### Development of targeted sequencing panel

We utilized the results of several studies to develop a panel of 122 genes for sequencing on an Ion Torrent PGM sequencer (Table 2). An effort was made to select genes from a variety of supercontigs and the final panel contained genes from 78 supercontigs. The sequence for each gene, along with 150bp of flanking sequence on both ends, was copied from VectorBase and formatted as a FASTA file. If there were small (< 100 bp) introns between exons, they were included for sequencing as well. One FASTA file per gene was submitted to Life Technologies’ AmpliSeq Designer (www.Ampliseq.com), where two pools of primers were designed that amplified 200 bp regions of each target gene. The two primer pools amplify overlapping segments of DNA, which increases the chances the entire gene of interest will amplify. For this study, a total of 1255 primer pairs (628 in Pool 1 and 627 in Pool 2) were designed.

**Table 2:** Names and sources of *Culex quinquefasciatus* genes included in the targeted sequencing panel.

SC	Gene ID	Name from Vectorbase	Source^a^	Comments
1	CPIJ000049	carboxylesterase	A	Culex expansion gene^b^
1	CPIJ000050	carboxylesterase	A	Culex expansion gene
1	CPIJ000051	carboxylesterase	A	Culex expansion gene
2	CPIJ000304	GST D2	A	Culex expansion gene
9	CPIJ000926	cytochrome P450	C	upregulated
12	CPIJ001038	cytochrome P450 CYP18A1	E	
12	CPIJ001081	beta, beta-carotene 15,15'-monooxygenase	E	
15	CPIJ001240	cathepsin B-like thiol protease	B	upregulated
25	CPIJ001746	conserved hypothetical protein	D	downregulated
25	CPIJ001759	cytochrome P450 CYP4H40	C	upregulated
23	CPIJ001836	cuticle protein	D	downregulated
21	CPIJ002128	mast cell protease 2 precursor	B	upregulated
21	CPIJ002130	kallikrein-7 precursor	B	upregulated
21	CPIJ002135	trypsin alpha-4 precursor	B	upregulated
35	CPIJ002538	cytochrome P450 CYP6AG12	C, B	upregulated
42	CPIJ002629	sensory appendage protein, putative	D	downregulated
36	CPIJ002663	Glutathione S-transferase 1–1	A	
36	CPIJ002678	Glutathione transferase I	A	Culex expansion gene
36	CPIJ002679	Glutathione S-transferase theta-2	A	Culex expansion gene
36	CPIJ002680	Glutathione S-transferase	A	Culex expansion gene
36	CPIJ002681	Glutathione S-transferase	A	Culex expansion gene
46	CPIJ002809	conserved hypothetical protein	D	downregulated
39	CPIJ003082	cytochrome P450 CYP9J42	C	upregulated
50	CPIJ003558	deoxyhypusine hydroxylase	E	
53	CPIJ003623	coagulation factor XII precursor	B	upregulated
60	CPIJ004086	angiotensin-converting enzyme	B	upregulated
73	CPIJ004532	40S ribosomal protein S17	F	Aedes housekeeping gene ortholog
86	CPIJ005332	cytochrome P450 CYP9J43	C	upregulated
104	CPIJ005953	cytochrome P450 CYP6BB3	B, C	upregulated
104	CPIJ005954	cytochrome P450 CYP6CC2	B, C	upregulated
104	CPIJ005955	cytochrome P450 CYP6P14	B, C	upregulated
104	CPIJ005956	cytochrome P450 CYP6BZ2	B, C	upregulated
104	CPIJ005957	cytochrome P450 CYP6AA9	B, C	upregulated
104	CPIJ005959	cytochrome P450 CYP6AA7	B, C	upregulated
106	CPIJ006034^c^	acetylcholinesterase	G	
106	CPIJ006067	ATP synthase B chain, mitochondrial	D	upregulated
106	CPIJ006068	utp-glucose-1-phosphate uridylyltransferase 2	D	upregulated
109	CPIJ006160	Glutathione s-transferase	B	upregulated
121	CPIJ006542	chymotrypsin-2	B	upregulated
156	CPIJ006721	cytochrome P450 CYP4H37	B	upregulated
139	CPIJ007047	serine/arginine rich splicing factor	D	downregulated
140	CPIJ007135	juvenile hormone esterase	A	Culex expansion gene
163	CPIJ007188	cytochrome P450 CYP4H30	B	upregulated
182	CPIJ007593	sodium channel protein para	G	
182	CPIJ007594	Voltage-dependent para-like sodium channel	G	
182	CPIJ007595	Sodium channel protein	G	
182	CPIJ007596	Voltage-gated sodium channel	G	
171	CPIJ007825	para-nitrobenzyl esterase	A	Culex expansion gene
196	CPIJ008566	cytochrome P450 CYP6Z15	C	upregulated
228	CPIJ009085	cytochrome P450 CYP6AG13	B	upregulated
241	CPIJ009106	angiotensin-converting enzyme precursor	B	upregulated
224	CPIJ009364^d^	anamorsin, putative	D	downregulated
229	CPIJ009404	trehalose-6-phosphate synthase	D	upregulated
234	CPIJ009415	cytochrome P450 CYP4G36	E	
247	CPIJ009474	cytochrome P450 CYP4D40	H	
247	CPIJ009478	cytochrome P450 CYP4D42	B, C	upregulated
240	CPIJ009578	CRAL/TRIO domain-containing protein	D	upregulated
252	CPIJ009715	putative uncharacterized protein	D	upregulated
279	CPIJ010227	cytochrome P450 CYP12F13	B, H, C	upregulated
261	CPIJ010238^d^	GLP_748_1200_211 (Fragment)	E	
277	CPIJ010480	cytochrome P450 CYP4J20	E	
278	CPIJ010537	cytochrome P450 CYP9J45	B, C	upregulated
278	CPIJ010538	cytochrome P450 CYP9J46	B, A	upregulated
278	CPIJ010543	cytochrome P450 CYP9J40	B, H	upregulated
278	CPIJ010544	cytochrome P450 CYP9J33	B, H, C	upregulated
278	CPIJ010546	cytochrome P450 CYP9J34	B, H, C	upregulated
278	CPIJ010548	cytochrome P450 CYP9J39	H, C	upregulated
281	CPIJ010805	carboxypeptidase A1 precursor	B	upregulated
293	CPIJ010814	Glutathione S-transferase 1–5	A	Culex expansion gene
332	CPIJ010858	cytochrome P450 CYP6F1	H	
313	CPIJ011127	cytochrome P450 CYP4H34	B, A	upregulated
328	CPIJ011693	NADH-ubiquinone oxidoreductase 42 kda subunit	D	upregulated
392	CPIJ012406^d^	histone cluster 1, putative	D	upregulated
392	CPIJ012466	pupal cuticle protein 78E, putative	D	downregulated
392	CPIJ012470	cytochrome P450 CYP9AL1	B, C	upregulated
435	CPIJ012935	thymosin isoform 1	D	downregulated
426	CPIJ013027	gut esterase 1 precursor	A	Culex expansion gene
448	CPIJ013319	metalloproteinase, putative	B	upregulated
469	CPIJ013503	NADH dehydrogenase iron-sulfur protein 3	D	upregulated
464	CPIJ013721	dimethylaniline monooxygenase	E	
464	CPIJ013723	dimethylaniline monooxygenase	E	
464	CPIJ013725	dimethylaniline monooxygenase	E	
512	CPIJ013917	esterase B1 precursor	G	esterase
512	CPIJ013918	esterase B1 precursor	G	esterase
510	CPIJ014218	cytochrome P450 CYP9M10	B, C	upregulated
561	CPIJ014523	elastase-3A precursor	B	upregulated
631	CPIJ015248	zinc-finger protein	H	
728	CPIJ015681	cytochrome P450 CYP4H37	H, B	upregulated
730	CPIJ015958	cytochrome P450 CYP325BC1	B, A	upregulated
832	CPIJ016012	tryptase-2	B	upregulated
729	CPIJ016026	carboxylesterase	A	Culex expansion gene
753	CPIJ016284	cytochrome P450 CYP4J4	E	
792	CPIJ016681	esterase FE4 precursor	A	Culex expansion gene
963	CPIJ017123	Myosin light chain 2	I	
970	CPIJ017198	cytochrome P450 CYP325BF1-de1b	E	
938	CPIJ017243	cytochrome P450 CYP304B4	B	upregulated
984	CPIJ017326	odorant binding protein OBP43	D	downregulated
977	CPIJ017331	cuticle protein CP14.6 precursor	D	upregulated
944	CPIJ017479	conserved protein, putative	D	upregulated
1030	CPIJ017763	juvenile hormone esterase precursor	A	Culex expansion gene
1170	CPIJ017894	voltage-gated sodium channel	J	
1170	CPIJ017895	voltage-dependent para-like sodium channel	J	
1170	CPIJ017896	voltage-gated sodium channel	J	
1118	CPIJ018232	cholinesterase	A	Culex expansion gene
1118	CPIJ018233	carboxylesterase	A	Culex expansion gene
1199	CPIJ018377	T-complex protein 1 subunit epsilon	D	upregulated
1224	CPIJ018624	Glutathione-s-transferase theta, gst	A	Culex expansion gene
1224	CPIJ018626	Glutathione-s-transferase theta, gst	A	Culex expansion gene
1224	CPIJ018627	Glutathione S-transferase 1–1	A	Culex expansion gene
1224	CPIJ018628	Glutathione S-transferase E2	A	Culex expansion gene
1224	CPIJ018629	Glutathione-s-transferase theta, gst	A	Culex expansion gene
1224	CPIJ018630	Glutathione S-transferase 1–1	A	Culex expansion gene
1224	CPIJ018631	Glutathione-s-transferase theta, gst	A	Culex expansion gene
1224	CPIJ018632^d^	Glutathione-s-transferase theta, gst	A	upregulated
1387	CPIJ018943	cytochrome P450 CYP4C52	C	upregulated
1643	CPIJ019395	cytochrome P450 CYP4C52	E	
2838	CPIJ019428	trypsin 2 precursor	B	upregulated
2060	CPIJ019703	cytochrome P450 CYP6Y6	E	
2176	CPIJ020030	fork head domain transcription factor slp2	I	
3121	CPIJ020082	cytochrome P450 CYP6F6	E	
2594	CPIJ020229	cytochrome P450 CYP4D42	B, C	upregulated

^a^A = [56], B = [48], C = [49], D = [50], E = Vectorbase search for monooxygenase genes, F = [62], G = Known insecticide resistance gene, H = Biomart search for *Culex* detoxification genes, I = [63], J = likely repeats of voltage-gated sodium channel gene.

^*b*^*Culex* expansion gene = as proposed by [55]

^c^Primers designed but no PCR products made for this gene.

^d^Primer design process was unable to find suitable primers for this gene so it was excluded.

### DNA extraction and sequencing library preparation

One mosquito each from 125 resistance-tested families was homogenized using a copper BB in 400 μl of BA-1 diluent on a TissueLyser homogenizer (Qiagen), and a 220 μl aliquot was used for DNA extraction on a BioRobot (Qiagen). Genomic DNA was eluted with 80 μl of AVE buffer and quantified using a Qubit 3 fluorometer (Life Technologies) with HS Assay reagents. Following quantitation, DNA was diluted with molecular biology grade water (Fisher Scientific) to 4 ng/ μl for making sequencing libraries.

We used the Ion AmpliSeq Library Kit 2.0 (Thermo Fisher) to create sequencing libraries following the manufacturer’s recommendations with the following modification: Instead of performing 20 μl initial PCR reactions with Hi-Fi mix, primers and DNA, we made two 10 μl reactions (10 ng DNA/reaction), one for each of the two primer pools. The two reactions were combined after the first PCR and preparation proceeded with the combined reactions. In order to sequence multiple individuals in one sequencing run, we barcoded each individual with Ion Express barcodes. Libraries were quantified using Ion Torrent’s TaqMan library quantitation kits, and diluted to 100 pM concentrations. A further dilution to 30 pM was performed prior to the beginning of template preparation, as per the manufacturer’s instructions.

We used an Ion Chef with HiQ and HiQ View reagents (Thermo Fisher) for template preparation and loading of the Ion Torrent semiconductor sequencing chips. Options for Chef runs such as kit type, barcode numbers and reference genome were selected using the Planned Run feature of our Ion Torrent server, the details of which were also used by the PGM sequencer. Barcoded libraries for 8–12 individuals were pooled for each 314 v2 BC sequencing chip by combining 3 μl of each 30 pM library. We found that the percentage of polyclonal beads was reduced when a volume of molecular biology grade water equal to 50% of the volume of the combined samples was added to the 30 pM sample before 25 μl was removed for the Chef run. The Chef prepared two sequencing chips per overnight run. Sequencing was performed on an Ion Torrent PGM sequencer using the previously established Chef planned runs and HiQ or HiQ View reagents. Two chips were sequenced successively, and the resulting sequencing files were exported as raw FASTQ files from the Ion Torrent server.

### Data analysis

The data analysis pipeline consisted of five parts: 1) reference generation, 2) quality assessment, trimming and filtering, 3) sequence alignment, 4) identification of CNV and SNP changes, and 5) feature selection based on comparing different sets of samples. Code for the pipeline is publicly available at (https://github.com/lkothera/cnv-snp-pipeline). The pipeline was designed to be generalized, allowing easy specification of samples and reference sequence in a configuration script. This flexibility enabled augmentation of analyses with additional samples, when desired, and did not hard-code the pipeline to a single mosquito species. For example, the pipeline was able to analyze sequencing data from another important vector mosquito species, *Aedes aegypti*, for a subsequent study. Once the configuration script was specified, the pipeline provided a command-line tool that allowed the user to run individual steps with a Unix-compatible “make” command.

The reference was generated using the CpipJ2 genome assembly available on VectorBase. Specifically, the reference consisted of the supercontigs that corresponded to the 118 genes in the panel and their associated gene coordinate information from VectorBase. This allowed exons to be accurately mapped for the purpose of SNP discovery. After reference generation, the FASTQ reads from the mosquito samples were trimmed and filtered using FaQCS and a quality threshold of 30 [64]. The resulting reads were then aligned by one of two methods, depending on the type of analysis. The BWA tool [65] was used for the CNV pipeline to align the filtered and trimmed sequences to the reference. For the SNP pipeline, a tool specifically for Ion Torrent data called TMAP was used (https://github.com/iontorrent/TMAP). TMAP is the preferred aligner for subsequent SNP calling by the OTG-snpcaller, again an Ion Torrent-specific tool [66]. FASTQ files of raw sequencing data (one per individual) were deposited with the Sequence Read Archive (SRA accession PRJNA533640).

The data were analyzed for two kinds of genetic changes, CNVs and SNPs. Although several tools are available to analyze next-generation sequencing data for variations in gene copy number, we chose CoNIFER (Copy Number Inference From Exome Reads) [67] because its algorithms are designed specifically for features of targeted sequencing data such as non-uniform read depths, systematic biases between sample runs, and rare variants. The analysis divided the samples into two groups based on resistance phenotype (resistant and susceptible) and determined the following for each gene: a fold change difference between groups, a t-test p-value, an mRMR (minimum redundancy, maximum relevance) ranking [68], and a determination of whether the gene was present in more (“duplicated”) or fewer (“deleted”) copy numbers. All genes had one or the other determination. Genes with both significant t-test p-values and a “duplicated” determination were suggested to be present in higher copy numbers in resistant vs. susceptible individuals.

Validation of the CNV results proceeded by designing primers and probes for real-time quantitative PCR reactions (S2 Table). Primer and probe sequences were designed using Primer Select (DNAstar/Lasergene). The ferritin gene (CPIJ004070) was used as a single copy reference gene [46]. All probes were 5’ labeled with FAM and 3’ labeled with BHQ1, and ordered from CDC’s Biotechnology Core Facility. A 20x primer-probe reaction mix was constructed for each candidate gene with the final concentrations of primers at 300 nM and probe at 200 nM. We used SsoAdvanced Universal Probes Supermix (Bio-Rad), which contained dNTPs, buffers and taq. Reactions were run in triplicate on a CFX-96 real-time thermal cycler (Bio-Rad) in single-plex 20 μl volumes using the manufacturer’s recommended cycling conditions: 2 minutes at 95°, followed by 40 cycles of 10 seconds at 95° and 30 seconds at 60°.

A standard curve was constructed for each primer-probe set to verify acceptable amplification at the range of concentrations expected during testing. After optimization, each candidate gene was assayed in a sample of resistant individuals (range = 24–42) drawn from among the eight populations in the study. The instrument’s analysis software calculated the fold-change of genomic DNA (gDNA) in candidate genes relative to the ferritin reference gene, using the CDC’s susceptible *Cx*. *quinquefasciatus* Sebring colony gDNA as the control. If the fold change of a gene was ≥ 1.5, it was taken as support that the gene was present in greater copy numbers in resistant individuals [69].

SNPs were called using a variation of the GATK (Genome Analysis Tool Kit) [70] pipeline for Ion Torrent data called *OTG-snpcaller* [66]. Individual Fisher’s Exact Tests were used to determine whether a SNP was significantly associated with a resistance phenotype. The GATK pipeline output was augmented with several descriptive characteristics for each SNP that aided with interpretation, including position in the supercontig and relative position within the codon. In addition, the location of the SNP relative to coding regions of genes was indicated, such as specifying that the SNP was in the flanking sequence, the gene itself, or in an intron.

The GATK pipeline output a set of SNPs (Data Set 1) that represented all SNPs in the study that passed the quality filters, including SNPs in genes, introns and the flanking sequences upstream or downstream of a gene. Data Set 1 was queried to identify significant SNPs relative to a particular set of resistant and susceptible individuals, a process called “feature selection”. In this way we were able to compare the resistant and susceptible phenotypes for each insecticide. We will hereafter refer to these data queries as “comparisons”.

The statistically significant SNPs for each comparison were retained by selecting those with Fisher Exact Test p-values ≤ 0.10, a threshold that was chosen because the test can be overly conservative [71] and SNPs would be subject to further analysis (see below). In all subsequent analyses, we kept only SNPs located in genes, disregarding SNPs located in flanking or intron sequences. This pruned data set (SNPs in genes, with Fisher Exact Test p-values ≤ 0.10) will be referred to as Data Set 2. Each list of significant SNPs (one list per comparison) was sorted by their AUC (Area Under the Curve; [72]) values. In this study, the AUC metric is an average of values the pipeline calculated for sensitivity and specificity. Those values are determined as follows:

sensitivity=#sampleswithresistantphenotypesANDalternatealleles/#sampleswithresistantphenotypes

specificity=#sampleswithsusceptiblephenotypesANDreferencealleles/#sampleswithsusceptiblephenotypes

Individual SNPs with higher AUC values are more informative compared to those with lower AUC values.

In order to further narrow down the number of informative SNPs, we then selected SNPs with p-values ≤ 0.05 for the Cochran-Armitage test for trend (CATT; [73, 74]). The CATT is a modified Pearson chi-squared test that in this context tests for the strength of the association between an alternate allele and the phenotype of resistance. A co-dominant model was applied to calculate the CATT and its associated p-value. We refer to these data (the subset of Data Set 2 SNPs with significant CATT p-values) as Data Set 3. From Data Set 3 we focused particularly on the SNPs that were nonsynonymous, as mutations that cause a change in amino acid composition may affect resistance status to a greater degree than synonymous ones. To examine whether there were population-level differences in the nonsynonymous SNPs from Data Set 3, we determined the frequencies of those SNPs in each population.

We were also interested in discerning the group of SNPs that were most associated with resistance. For this we utilized two parts of the R package *adegenet*: a Discriminant Analysis of Principal Components (DAPC), and the associated *snpzip* procedure [75]. Data Set 1, which contained all of the SNPs that passed quality filters, was used for this analysis. The data were pruned to remove SNPs from the analysis that were monomorphic (but different than the reference genome) and to remove SNPs with more than 5% missing data. The results were similar whether data were organized by malathion resistance phenotype or permethrin resistance phenotype, so we used the permethrin resistant phenotypes. The results of a DAPC are sensitive to the number of principal components retained, so a cross-validation bootstrapping (*xval*) was performed to determine the optimal number of principal components. One principal component was retained. The *snpzip* procedure identifies “structural” SNPs, i.e. the ones that contribute most to population structure, here defined as resistance phenotype. The results are returned as a list of SNPs with the highest loading scores.

## Results

### Resistance phenotypes and mutations in the VGSC gene

Although sequencing coverage in general was very good (50x or more) there was not perfect amplification of every gene submitted for primer design. In some instances there were gaps where no sequencing data were generated, presumably due to a failure of primers to adequately amplify their targets during the first step of library preparation. For example, no suitable primers could be designed for a few genes (n = 4 and noted in Table 2). In addition, there was a lack of sequence generated for the entire acetylcholinesterase gene CPIJ006034, wherein the primers apparently did not amplify their intended target regions. This resulted in a lack of data for this gene and therefore no data for the *ACE-1* mutation for these specimens. Finally, there were a few instances where sequences generated by the process mapped to genes not on the list submitted for primer design. Of these, three genes had SNPs in Data Set 3, but all changes were synonymous.

Even though our study design involved sampling egg rafts from areas that did not receive routine adulticide treatments, the majority of individuals sequenced were resistant to one or both insecticides (Table 3). Most families (N = 82) were resistant to both malathion and permethrin. The least common combination of phenotypes was resistance to malathion and susceptible to permethrin (N = 8). The L1014F *kdr* mutation in gene CPIJ007595 was common across all populations and the frequency of individuals having at least one copy ranged from 37% in the TXU population to 100% in the HCU population. All 1014 *kdr* mutations observed had the same nucleotide change: TTA > TTT. No other mutations (e.g. L1014S, or L1014H) were found at this locus. Nine wild type (SS) individuals had permethrin susceptible phenotypes, and 13 SS individuals had resistant phenotypes. No other previously described *kdr* mutations (i.e. those found in different parts of the VGSC gene) were statistically significant in the comparisons for either insecticide. While the L1014F *kdr* mutation was statistically associated with resistance when individuals were grouped by permethrin phenotype, the association was not statistically significant for the malathion comparison (Fisher Exact Test p = 0.77).

**Table 3:** Numbers and resistance phenotypes of the 125 *Culex quinquefasciatus* families in this study. Each family was split and tested for resistance to both insecticides. A single individual from each family was then sequenced.

Insecticide	# Susceptible	# Resistant
Malathion	35	90
Permethrin	19	106

The SNP analysis initially identified four highly significant SNPs in Exon 4 (as described by VectorBase) of gene CPIJ007596, which is adjacent to the gene with the L1014F *kdr* mutation, CPIJ007595, and thus of high interest in terms of a novel *kdr* mutation. However, attempts to perform reverse-transcription PCR (RT-PCR) using individual mosquito RNA to verify that Exon 4 was expressed indicated it was not transcribed, although Exons 3 and 5 were.

### Copy number variants (CNVs)

The CoNIFER results suggested 16 unique genes were amplified in resistant individuals compared to susceptible individuals (t-test p-value ≤ 0.05; Table 4). There was no overlap in the lists of genes between the two insecticides. With respect to increased copy number genes associated with malathion resistance, six GST genes located on the same supercontig figured prominently in the statistical analysis. However, GST genes were absent from the permethrin list, which had fewer genes overall (n = 5) and consisted of four P450 genes and the VGSC gene CPIJ007595.

**Table 4:** CoNIFER results showing genes (by insecticide) present in greater copy numbers (T-test p-value ≤ 0.05) in resistant vs. susceptible individuals.

Insecticide	Gene ID	Gene Type or Gene Family^a^	Experimentally validated with real-time PCR	Evidence of increased copy number
Malathion	CPIJ003082	P450	x	x
	CPIJ006160	GST	x	
	CPIJ013319	metalloproteinase, putative	x	x
	CPIJ017479	unknown		
	CPIJ018377	Chaperonin	x	x
	CPIJ018624	GST	x	
	CPIJ018626	GST		
	CPIJ018627	GST		
	CPIJ018628	GST	x	
	CPIJ018629	GST		
	CPIJ018630	GST	x	x
	CPIJ007595	VGSC	x	x
Permethrin	CPIJ010548	P450		
	CPIJ010543	P450	x	x
	CPIJ018943	P450		
	CPIJ019395	P450	x	

^a^GST = Glutathione S-Transferase, P450 = Cytochrome P450, VGSC = Voltage Gated Sodium Channel

**Fig 2:**
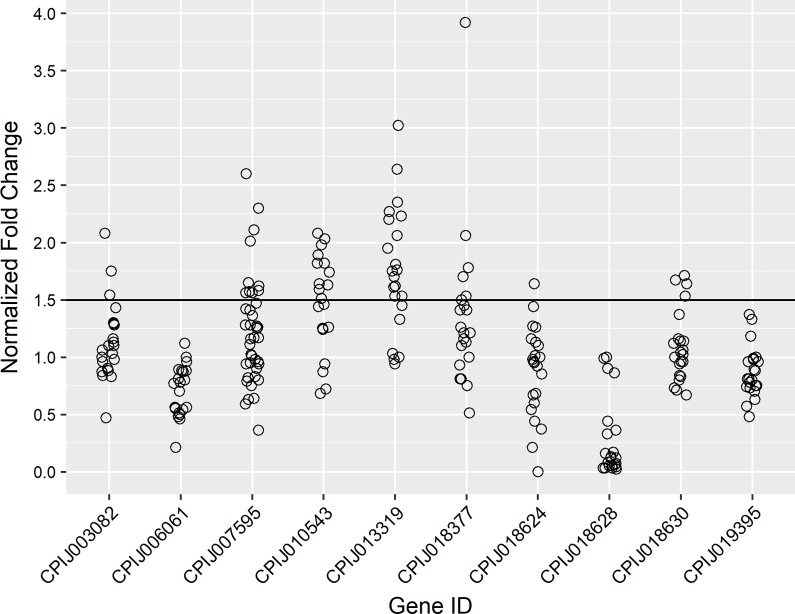
Chart showing CNV (copy number variation) validation results with gDNA as normalized fold changes of candidate genes in phenotypically resistant individuals. Each open circle represents one individual mosquito. Fold-changes ≥ 1.5 were taken as evidence of increased copy number.

We experimentally validated the CNV results for 10/16 of the genes listed in Table 4. Quantitative Real-Time PCR results suggested a total of six genes were present in over 1.5 copies in some resistant individuals: a GST gene (CPIJ018630), two P450 genes (CPIJ003082, CPIJ010543), the VGSC gene (CPIJ007595), a metalloproteinase gene (CPIJ013319) and a Chaperonin gene (CPIJ018377; Fig 2). Individuals tested against the remaining genes did not display fold changes consistent with increased copy numbers. With respect to the VGSC gene, our initial validation assays included individuals from the following populations: HCT, NOT, TXT and TXU. Interestingly, only the TXU and TXT populations displayed 1.5 or more fold changes for the VGSC gene, despite many individuals having two copies of the L1014F *kdr* mutation. An additional validation assay was run with gDNA from the TXU and TXT populations, and greater than 1.5 fold copy numbers were observed for three permethrin susceptible TXU individuals *without* the *kdr* mutation (i.e. wild type) and for five resistant TXT individuals with two copies of the *kdr* mutation.

### SNP analysis results

A total of 5,940 SNPs were identified after QC efforts and comprise Data Set 1. Of this number, 3,769 were in genes, 1,076 were located in the flanking regions before or after a gene, and 1,095 were located within introns. Data Set 2 had 571 SNPs (with Fisher Exact Test p-values ≤ 0.10) between the two comparisons. A total of 80 SNPs (14% of the total) were found in both comparisons, and the remaining SNPs were associated with either malathion or permethrin resistance. These data are sorted by AUC values, and presented inS3 Table.

Data Set 3 (SNPs associated with significant p-values for the Fisher Exact Test and the CATT) was comprised of 228 unique SNPs across the two comparisons: 144 SNPs in 39 genes for malathion and 84 SNPs in 26 genes for permethrin (S4 Table). There were 22 SNPs significantly associated with both insecticides (12% of the total), 18 of which were in the two esterase genes CPIJ013917 and CPIJ013918. With respect to the gene families represented by Data Set 3, cytochrome P450 genes were present over twice as often as two other gene families commonly associated with resistance (GSTs and Esterases). Specifically for the malathion comparison, there were significant SNPs in 18 P450 genes, seven GST, five esterase genes, and eight genes from other families for a total of 39 genes. Ten genes (56%) were on the list of “expansion” genes from Yan et al. [55]. For the permethrin comparison, there were 13 P450 genes, four GST and three esterase genes represented. The number of SNPs per gene ranged from 1–16 in the malathion comparison and 1–25 in the permethrin comparison. Indeed, the 25 SNPs in the P450 gene CPIJ010544 accounted for 30% of the permethrin SNPs in Data Set 3. There were four genes (15%) associated with permethrin resistance in Data Set 3 from the “expansion” genes [55].

We examined Data Set 3 to determine the frequency of synonymous and nonsynonymous SNPs. In the malathion comparison, 93/144 of SNPs (64.5%) were synonymous, 50/144 (34.7%) were nonsynonymous and at one locus two changes were observed, one synonymous, one nonsynonymous. A relatively larger proportion of SNPs were nonsynonymous in the permethrin comparison, 77.4% (65/84). The nonsynonymous SNPs shared between the two comparisons were in esterase genes CPIJ013917 and CPIJ013918 (n = 6).

**Fig 3:**
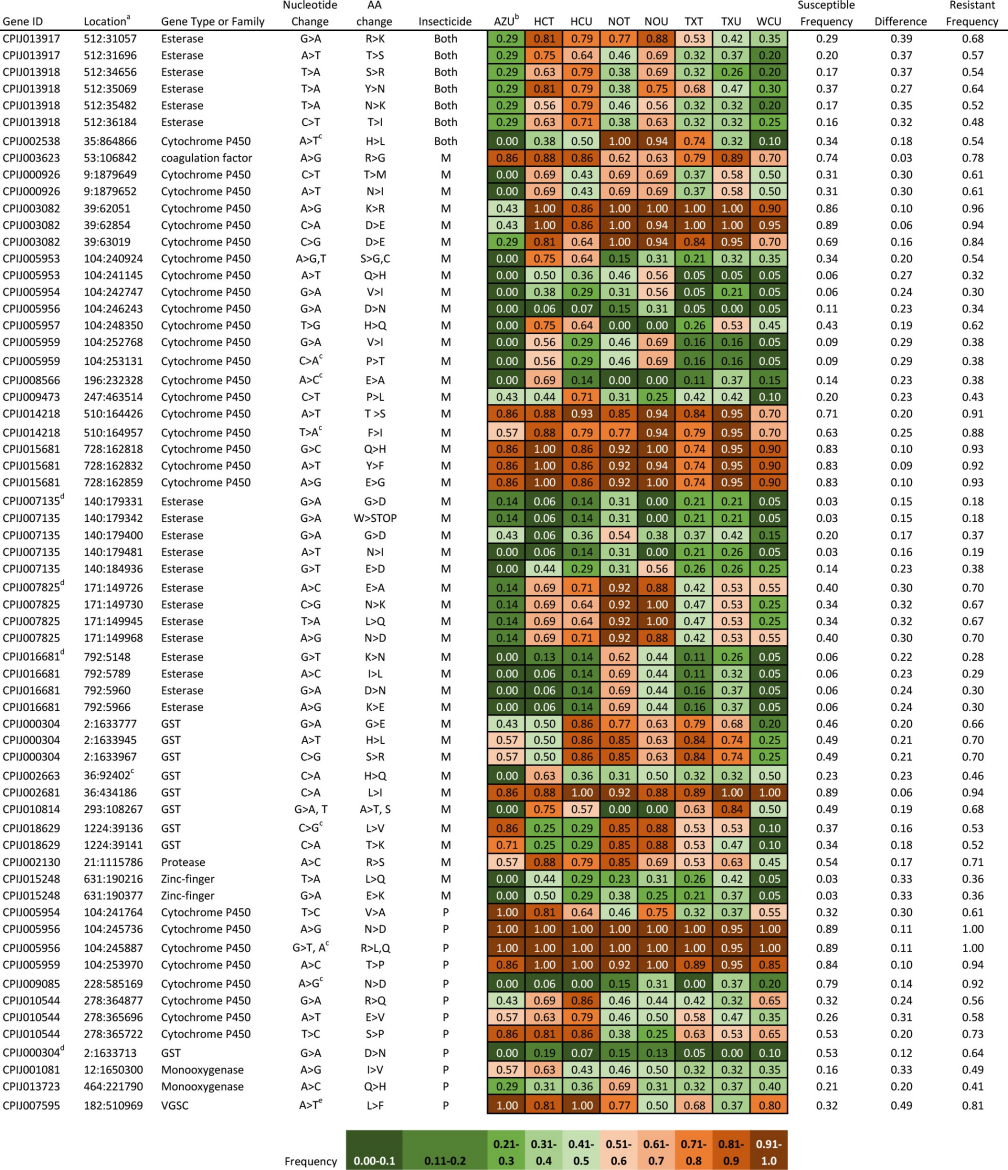
Non-synonymous SNPs (listed as supercontig:position) associated with resistance to malathion (M), permethrin (P), and both insecticides, as well as SNP frequency per population and frequency of each SNP in susceptible vs. resistant phenotype individuals overall. ^a^Position of significant SNPs in Data Set 3 relative to the supercontig. ^b^See Table 1 for population information. ^c^SNP occurs in functionally conserved region of the gene (See Table 5). ^d^"expansion" gene as per Yan et al. 2012. ^e^This is the L1014F kdr mutation.

Fig 3 lists the all of the nonsynonymous SNPs from Data Set 3, their frequencies per population, and their frequencies across susceptible and resistant individuals. Cytochrome P450 genes were the most common gene type possessing nonsynonymous SNPs statistically associated with resistance in our study. Esterase and GST genes were also represented, as were a small number of other types of genes. We include also in Fig 3 the *difference* between a SNP’s frequency in resistant individuals vs. its frequency in susceptible individuals. In several instances, even though the results were statistically significant, only small differences in SNP frequencies were observed because almost all individuals, regardless of phenotype, possessed the SNP, relative to the reference. Examples of such SNPs occurred in genes CPIJ005956 and CPIJ015681. The largest difference in SNP frequency between phenotypes was the L1014F *kdr* mutation, where 81% of resistant individuals had at least one copy of the SNP, and 32% of susceptible individuals had at least one copy, for a difference of 0.49. The remaining differences ranged from 0.39–0.03. Several SNPs were present in low to moderate frequencies (0.05–0.31) in all populations, for example in the gene CPIJ007135. Of greater interest in terms of SNPs potentially useful as diagnostic markers, several SNPs were present in relatively few susceptible individuals and also in most resistant individuals. An example of such SNPs were in the gene CPIJ015248, which codes for a zinc finger protein. Population frequencies for this mutation were low in two populations (AZU and WCU) and moderate in the remaining populations. The difference in the frequency of the mutations in this gene between susceptible and resistant individuals overall was 33%. Genes that showed a similar pattern of low frequency in susceptible and moderate frequencies in resistant individuals included CPIJ007825, an esterase gene described as an “expansion” gene by Yan et al. [55], and CPIJ005959 (*CYP6AA7*), a P450 gene.

Frequencies of SNPs in Data Set 3 varied considerably at the population level despite a large proportion of each population being resistant to one or both insecticides. As mentioned above, although statistically significant, some SNPs were present in high frequencies in both susceptible and resistant individuals, and this was reflected in high frequencies in all populations. However, often the SNPs in Fig 3 occurred in high frequencies in some populations but not others. For example, most of the individuals in the Harris County populations (HCT and HCU) and the New Orleans populations (NOT and NOU) were resistant, and had relatively high frequencies of Data Set 3 SNPs. Populations with more susceptible individuals (TXT, TXU and WCU) generally had lower frequencies of the same SNPs. A few SNPs seemed to be present in high frequencies in only one or two populations, such as those in CPIJ016681, where 62%-69% of NOT individuals had the SNP, but frequencies range from 0–37% in the other sampled populations. Population WCU was notable for having an overall lower frequency of Data Set 3 SNPs compared to other populations.

Codon changes relative to the *Culex quinquefasciatus* reference genome (Fig 3) were examined using a BLAST search to determine whether they were located in functionally conserved regions. Protein BLAST [76] was used for genes in the GST and esterase gene families, which identified the positions of conserved residues and provided additional information as to their function. The codon positions of regions corresponding to cytochrome P450 gene Substrate Recognition Sites (SRS) 1–6 [77] were determined with a CYPED (CYtochrome P450 Engineering Database; [78]) BLAST search, which was then compared to the codon changes in Fig 3. Eight nonsynonymous SNPs, each in a different gene, appear to be located in conserved, functional regions of genes (Table 5).

**Table 5:** Nonsynonymous SNPs located in positions that contribute to secondary structure of the protein.

Gene ID	Location^a^	Reference codon	Alternate codon	Amino Acid Number	Stuctural features affected by SNP^b^	Insecticide	Gene Type or Family
CPIJ002538	35:864866	CAT	CTT	293	SRS3	Both	Cytochrome P450
CPIJ002663	36:92402	CAC	CAA	101	H-site	Malathion	GST
CPIJ005956	104:245887	CGA	CTA,CAA	213	SRS1	Permethrin	Cytochrome P450
CPIJ005959	104:253131	CCA	ACA	215	SRS1	Malathion	Cytochrome P450
CPIJ008566	196:232328	GAA	GCA	243	SRS2	Malathion	Cytochrome P450
CPIJ009085	228:585169	AAC	GAC	211	SRS1	Permethrin	Cytochrome P450
CPIJ014218	510:164957	GAA (TTC)^c^	GAT (ATC)	245	SRS2	Malathion	Cytochrome P450
CPIJ018629	1224:39136	TAG (CTA)	TAC (GTA)	207	H-site	Malathion	GST

^a^Position relative to the supercontig.

^b^SRS = Subtrate Recognition Site. H-site is part of the substrate binding pocket. See text for details.

^c^Codons in parentheses are the relevant codons when the opposite strand was sequenced.

The *snpzip* analysis in *adegenet* identified a total of 65 SNPs on two supercontigs that were most associated with the permethrin resistant phenotype (Table 6). The SNPs were located in two groups of sequentially numbered genes, CPIJ005954-CPIJ005959 and CPIJ010544-CPIJ010546. Genes CPIJ005958 and CPIJ010545 were not included in the study. By far, the gene with the most SNPs in the *snpzip* analysis was CPIJ010544, with 27 SNPs, followed by CPIJ005955 with 10.

**Table 6:** Results of *snpzip* analysis showing genes with SNPs that were the most informative for separating permethrin resistant and susceptible phenotypes.

Gene	SC^a^	No. SNPs
CPIJ005954	104	4
CPIJ005955	104	10
CPIJ005956	104	7
CPIJ005957^b^	104	5
CPIJ005959	104	7
CPIJ010544	278	27
CPIJ010546	278	5
	Total	65

All SNPs in the permethrin list were found in the malathion list, which itself had 19 unique SNPs.

^a^SC, Supercontig number in *Cx*. *quinquefasciatus* genome.

^b^Included in the SNPs for this gene are two SNPs in its upstream flanking sequence.

## Discussion

The case for identifying and validating genetic markers to characterize resistance with the goal of developing better diagnostic tools has been made recently for *Anopheles* [79] and *Aedes* [53] mosquitoes and for arbovirus vectors in general [80, 81]. Studies by Liu and colleagues on *Culex quinquefasciatus* [18, 47–49, 52, 82] have laid the groundwork to reduce the number of candidate genes potentially involved in insecticide resistance in this species. Their work has confirmed that a large number of genes with different functions are upregulated or downregulated in response to insecticide exposure or insecticide selection. A distinction between that body of work and our project is our emphasis on SNP and CNV differences, as opposed to describing differential gene expression, a decision made to work towards the goal of using gDNA from field-caught specimens to characterize insecticide resistance.

In this study we sequenced a panel of 122 genes in 125 unrelated *Culex quinquefasciatus* individuals. Our study design intentionally differed from some other vector mosquito next-generation sequencing studies, which generally use pooled DNA of colony individuals. Instead, we used families from field-collected egg rafts to determine resistance phenotypes and then used one individual per family to conduct DNA sequencing. Moreover, we sampled different populations to ascertain whether genetic changes associated with resistance to malathion or permethrin were consistent among populations or if there were among-population differences.

Although the primers for a small number of genes did not generate sequence data, overall the process generated sufficient coverage for data analyses. In particular, it would have been instructive to see the frequency with which the *ACE-1* mutation in gene CPIJ006304 occurred with the *kdr* mutation, but there was no sequence data generated for that gene. Berticat et al. [83] proposed that possessing the two above mutations could have synergistic effects on the resistance status of *Culex* mosquitoes, but stressed it was difficult to predict what the effects would be. The failure of some primers to amplify an important target highlights a drawback of using pools of primers to amplify genes for targeted sequencing. Changing the primers for one gene would require reformulating the entire set of over 1,000 primers, which was cost-prohibitive.

As expected, the *kdr* mutation was associated with resistance to permethrin, and not associated with resistance to malathion. While examining the SNP analysis output, we noted a discrepancy between the sequence for one of the VGSC genes (CPIJ007956) in the published *Cx*. *quinquefasciatus* genome in VectorBase, and sequences denoted as “*Culex* Sodium Channel mRNA” in GenBank. In particular, VectorBase lists an exon (Exon 4) for CPIJ007596 whose expression we attempted to confirm through RT-PCR but were unable to do so. A sample of *Culex quinquefasciatus* VGSC GenBank sequences (e.g. accession numbers BN001090.1, EU817515.1, JN695777, and KC977455.4) also do not list Exon 4 as part of the VGSC. For these reasons, it appears that what is listed as Exon 4 is in fact intron sequence. As mentioned previously, genes in the *Cx*. *quinquefasciatus* genome have not been fully mapped to chromosomes, although preliminary work was performed by Dudchenko et al. [59] who assembled chromosome-length scaffolds for the *Cx*. *quinquefasciatus* genome alongside the genome they were studying, *Aedes aegypti*. There is a need to complete the work of validating gene locations for the *Culex quinquefasciatus* genome, which will likely reduce the number of inconsistencies and mistakes in the current build and allow for better analyses at the genome level.

### Copy number variants

The qPCR assays designed to validate the CNV analysis results suggested several genes are present in multiple copies, as determined by the presence of at least some resistant individuals displaying ≥ 1.5-fold difference compared to the susceptible Sebring strain. Although the CNV analysis suggested a group of GST genes (CPIJ108624-CPIJ018630) were present in greater copy numbers, only one of the three genes assayed from this group displayed any evidence of amplification. The gene, CPIJ018630 (*GSTE2*) is an orthologue to gene AAEL007951, which was also observed to be present in multiple copies in *Aedes aegypti* targeted sequencing work [84]. The metalloproteinase gene CPIJ013319 had the greatest proportion of tested individuals showing increased copy numbers versus the susceptible control. This gene has orthologues in several species of mosquitoes including *Aedes aegypti*, but was not on a list of amplified genes in the targeted sequencing work mentioned above.

Gene amplifications in the *Cx*. *quinquefasciatus* VGSC gene have been previously described in a small number of studies [16, 85]. Our validation results were intriguing because the only populations showing evidence of VGSC gene amplification were from the Dallas, TX area (TXT and TXU). Further, we observed that some susceptible individuals without the L1014F mutation appeared to have multiple copies of the gene, suggesting the gene duplication event may predate the appearance of the mutation.

Finally, our CNV validation results indicated that two different P450 genes appeared to be present in multiple copies in individuals resistant for each insecticide. The one associated with malathion resistance, CPIJ003082 did not have a corresponding orthologue shown to be amplified in targeted sequencing work on *Aedes aegypti*, but the gene associated with permethrin resistance, CPIJ010543 (*CYP9J40*) did, in gene AAEL014619 (*CYP9J22*) [84].

A recent paper by Weetman et al. [86] makes the case that CNV is an important means by which mosquitoes become resistant, and that as detection methods evolve, the observed instances of CNV related to resistance will likely increase. In some instances, possessing two or more heterozygous copies of a resistance allele is thought to offset fitness costs associated with possessing important target site mutations. Weetman et al. also discussed amplifications in the esterase genes CPIJ013917 and CPIJ013918, referred to as *Est2* and *Est3*, respectively. Work dating back to the 1980s has tracked frequencies of amplified esterase alleles in *Culex pipiens* populations, mostly outside of the U.S. The link between resistance and gene amplifications has been well established in European *Culex pipiens* complex populations [41] but the CNV analysis performed by Conifer on our samples did not identify either esterase gene as being present in multiple copies. Reasons for this disparity could be due to the nature of targeted sequencing and subsequent analysis. Primers are designed to amplify 200bp regions of the gene and due to sequence polymorphisms some areas within the same gene may amplify better than others. The Conifer analysis averaged the sequencing reads across genes and normalized reads across individuals to make a determination of whether a gene was present in multiple copies, so it is possible that poor amplification over one or more parts of either esterase gene could appear to the analysis as a lower number of reads overall. It is also possible that the genes are duplicated in enough susceptible individuals that the analysis did not find resistant individuals were significantly different in terms of copy number.

### SNP analysis

Our study found a greater number of SNPs significantly associated with malathion resistance than with permethrin resistance. It is possible that sample numbers played a role in this result, because the proportion of susceptible to resistant individuals for the malathion comparison (35/90) was not as uneven as that for the permethrin comparison (19/106). It is also possible that the ubiquity of the *kdr* mutation in these populations has resulted in a selective sweep that has effectively reduced the ways that genes in permethrin susceptible individuals are different from resistant ones. An examination of the CATT p-values associated with individual SNPs in Data Set 3 (S4 Table) may support this assertion. For example, the L1014F *kdr* mutation has by far the smallest CATT p-value (0.000001). The next most significant SNPs have CATT p-values around 0.0005, followed by 28 SNPs with CATT p-values around 0.001. In contrast, there are more highly significant SNPs associated with malathion resistance. For example, there are 14 SNPs with CATT p-values around 0.00001, and 16 with CATT p-values around 0.0005.

Furthermore, the kinds of genes in which significant SNPs were found was different for each insecticide. That esterase genes, either by upregulation or duplication in the genome are capable of detoxifying organophosphate insecticides such as malathion, has been known for some time [87]. Our finding that additional esterase genes besides CPIJ013917 and CPIJ013918 are significantly associated with malathion resistance is therefore somewhat expected, but also underscores the value of a wider sequencing approach when studying resistance.

We examined the mutations in gene CPIJ015248, a zinc-finger protein to see if they occurred in or near conserved domains. One, an E>K change at nucleotide 190377 is between two zinc binding site Cysteine residues. The mutation is present in all sampled populations and occurred in 36% of resistant individuals. Conversely only one susceptible individual had the mutation. This gene is not well characterized and represents a potential candidate for future validation studies to explore its role in resistance.

Almost all of the genes that had SNPs significantly associated with both insecticides were the previously-mentioned esterase genes CPIJ013917 and CPIJ013918 (n = two and four SNPs, respectively). This finding could represent the importance of the two genes to detoxification in general in *Culex quinquefasciatus* mosquitoes. There is a fair degree of variation in the population frequencies of the six SNPs (Fig 3), with three of the four highly resistant populations (HCT, HCU and NOU) showing high frequencies, but the fourth population (NOT) showing moderate frequencies.

When we compared the positions of codons altered due to nonsynonymous SNPs (Fig 3) to the positions of known conserved functional regions of three gene families (esterase, GST, and cytochrome P450) we observed eight instances where the SNP occurred in such a region. No esterase genes showed alterations to functional regions. Taken with the above paragraph, this finding supports the idea that the esterase genes in this study are broadly important for detoxification. However, nonsynonymous SNPs caused codon changes in functional parts of two GST and six cytochrome P450 genes involving both insecticides (Table 5). In the GST genes, the codon substitutions were in the H-site, which binds substrates that are hydrophobic. The codon changes observed in functional regions of cytochrome P450 genes occurred mostly in SRS1, with one instance each of a codon change in SRS2 and SRS3. Such codon changes have been demonstrated to affect insecticide metabolism in other insects [53, 88] and represent a potential area of expanded investigation in *Culex quinquefasciatus*.

With respect to permethrin resistance, there were 25 significant SNPs located in gene CPIJ010544, more by far than in any other gene we sequenced. Fig 3 shows the population frequencies for the three nonsynonymous SNPs (range 25–86%) in this gene. No SNPs from this gene are significantly associated with malathion resistance, when the CATT p-value ≤ 0.05 cutoff was used. Such a large number of SNPs in one gene may be useful for developing a diagnostic assay, should this gene be shown to affect levels of resistance after further validation. On a related note, the large number of significant SNPs in CPIJ010544 was reflected in the *snpzip* analysis, although it indicated SNPs on a nearby gene (CPIJ010546) and a cluster of genes on another supercontig were also important in discriminating between resistant and susceptible phenotypes. Further, the *snpzip* analysis suggests a number of SNPs to investigate in terms of whether they, as a set, or group, are good at predicting insecticide resistance (Table 6).

A number of cytochrome P450 genes are overexpressed in insecticide resistant *Cx*. *quinquefasciatus* specimens, indicating they are involved in metabolic resistance either with or without the presence of the L1014F *kdr* mutation. For example, other studies conducted on specimens from Alabama indicated the following P450 genes were found to be upregulated in resistant strains: CPIJ018943 (*CYP4C52v1*), CPIJ010546 (*CYP9J34*), CPIJ005955 (*CYP6P14*), CPIJ010543 (*CYP9J40*), CPIJ014218 (*CYP9M10*), and CPIJ005959 (*CYP6AA7*) [47, 18]. Delanny et al. [44] examined CPIJ011127 (*CYP4H34*) and CPIJ010537 (*CYP9J45*) in addition to a similar set of P450 genes. They found a comparable pattern of upregulation in *Culex quinquefasciatus* specimens, although the latter were collected from Guadeloupe Island in the French West Indies, over 3,000 km away. Interestingly, as in our study, they observed population-level differences in the genetic differences they measured, adding support to the idea that selection for resistance can follow distinct trajectories in different populations. Our work showing SNP differences in many of the same genes suggests that sequence differences could contribute to observed differences in gene expression.

The gene CPIJ014218 (CYP9M10) has received attention for its relationship to insecticide resistance. Hardstone et al. [89] and Itokawa et al. [90] have described expression level differences, duplication events, and SNP differences in this gene between resistant and susceptible strains of *Culex quinquefasciatus*. The SNP differences were not the same as the ones we found, again suggesting that different outcomes may result when populations are under selective pressure from insecticide application. Itokawa et al. [91] used the gene-editing technologies TALEN (transcription activator-like effector nucleases) and CRISPR to knock out CPIJ014218 and found a reduction in levels of resistance in tested specimens.

## Conclusions

An international symposium on insecticide resistance in mosquito vectors [81] stressed the importance of developing novel tools and strategies to detect and manage resistance. In a synopsis on how to improve insecticide resistance surveillance, they noted that in regards to target site mutations “Molecular diagnostics are currently underused for predictive purposes, and with DNA-based tests applicable to almost any samples, marker-based assays present great potential to yield fine-scaled data.” They go on to state, “Functional validation of DNA markers for most metabolic resistance mechanisms is also a priority to speed up the implementation of resistance management strategies”.

Studies such as ours contribute to efforts to develop molecular markers to characterize insecticide resistance in *Culex pipiens* complex mosquitoes. We identified a number of statistically significant genetic changes associated with the resistance phenotype by using targeted DNA sequencing of a panel of 122 genes. Future work should be on two fronts: 1) functional validation of significant SNP and CNV changes observed in this study and 2) a broadening of the sequencing approach to include whole genome and/or transcriptome sequencing of resistant and susceptible individuals. In doing the latter we expect to gain insight into a larger set of genes that may be important to characterizing resistance. Work on *Aedes aegypti* indicated there were multiple means by which resistant individuals differed from susceptible ones, including sequence polymorphisms and copy number variants, but intriguingly also described instances where sequence polymorphisms in promoter regions upstream of gene sequences were found to be associated with gene upregulation in resistant specimens [53]. Such polymorphisms could provide a means to develop PCR-based diagnostic tests for metabolic resistance that at present to not exist for *Culex* mosquitoes. Having such data, particularly for different *Culex pipiens* complex populations, will contribute greatly to developing the means to use molecular diagnostics to characterize insecticide resistance.

## Supporting information

S1 Table Resistance phenotypes for 125 families tested with the CDC bottle bioassay. (XLSX)

S2 Table Primer and probe sequences used to validate CNV determinations. All sequences listed 3'-5'.
(XLSX)

S3 Table Data Set 2 SNPs for Malathion (first sheet) and Permethrin (second sheet) sorted by AUC. (XLSX)

S4 Table Data Set 3 SNPs for Malathion (first sheet) and Permethrin (second sheet), sorted by Cochran Armitage Test for Trend (CATT) p-value. (XLSX)
